# Off-pump CABG procedures and mediastinitis: report on 723 consecutive patients

**DOI:** 10.1186/1749-8090-10-S1-A259

**Published:** 2015-12-16

**Authors:** Kosmas Tsakiridis, Nikos Sachpekidis, Andreas Mpakas, Alexandros Kolettas, Stamatis Arikas, Giorgos Tagarakis, Pavlos Zarogoulidis

**Affiliations:** 1 Cardiac & Thoracic Department, St.Luke's Private Hospital, 55236, Panorama, Thessaloniki, Greece

## Background/Introduction

Sternal wound complications occur rarely but sometimes are devastating for the patients. Although appropriate therapy mediastinitis also can lead to death in some patients.

## Aims/Objectives

To present our results of 723 consecutive CABG procedures in regard to the frequency of mediastinal infections/complications.

## Method

We included in the study 723 patients submitted to elective CABG-off pump procedure in the 15 year-period 2000-2014. The characteristics of our sample included: 498 male and 225 female patients. Mean age was 68,7 ± 6,9 years. The vast majority of patients (644-89%) were submitted to off-pump procedure, mostly with skeletonized bilateral internal thoracic artery (BITA) revascularization. Care was taken that the patients were extubated and transferred to the ward early. Mean discharge period was 7.2 days. Subgroups with extra risk factors for sternal/mediastinal complications such as diabetics or patients with COPD, BITA and obesity were provided with thoracic bandage and advised to avoid extra tension in the area.

## Results

No cases of mediastinal infection were observed; in total, only 51 cases (7%) of superficial sternal wound infection were noted.

## Discussion/Conclusion

Off pump-CABG procedures can be safely performed in regard to mediastinal complications, even in subgroups of patients with augmented risk for mediastinitis.

